# Art Must Be Free

**Published:** 1892-06

**Authors:** 


### ART MUST BE FREE.

The opening of the first National Art Congress recently at the Smithsonian Institution, in Washington, is the most important official step the artists of this country have ever taken.

They have assumed the national standing from a political standpoint, which is their right and which their artistic importance most fully warrants.

It is to America that the whole world looks for the great school of art of the future, and now that its foundation stones are so securely laid the architects and builders fitly justify their standing before the country.

They have supreme right to be heard on the question of free art, and that the great majority demand the abolishment of all duty should insure that this should be the law.

Senator Walcott, of Colorado, in pledging himself to mend his ways and work for free art, made the pertinent suggestion that some of the alleged art around Washington be removed at the same time with the duty. Then Governor McKinley has pledged himself to do all in his power for the advancement of art, and Secretary of War Elkins made the suggestion that Congress authorize the appointment of a committee of artists to choose all designs of monuments for the government.

